# Genome Sequence of *Gordonia terrae* Bacteriophage Wheezy

**DOI:** 10.17912/micropub.biology.001407

**Published:** 2025-01-07

**Authors:** Rachel M. Heyne, Catherine P. Chia

**Affiliations:** 1 School of Biological Sciences, University of Nebraska–Lincoln, Lincoln, Nebraska, United States

## Abstract

Bacteriophage Wheezy, a lytic phage with siphoviral morphology isolated using the host *Gordonia terrae* 3612, has a genome of 67,021 base pairs and is 65.9% GC. The genome sequence of Wheezy aligns most closely with subcluster CR2 phages Tracker and NatB6. Annotation of the full-length genome sequence of Phage Wheezy revealed 92 protein-coding genes and no tRNA genes.

**Figure 1. Transmission electron micrograph of Wheezy virions:**
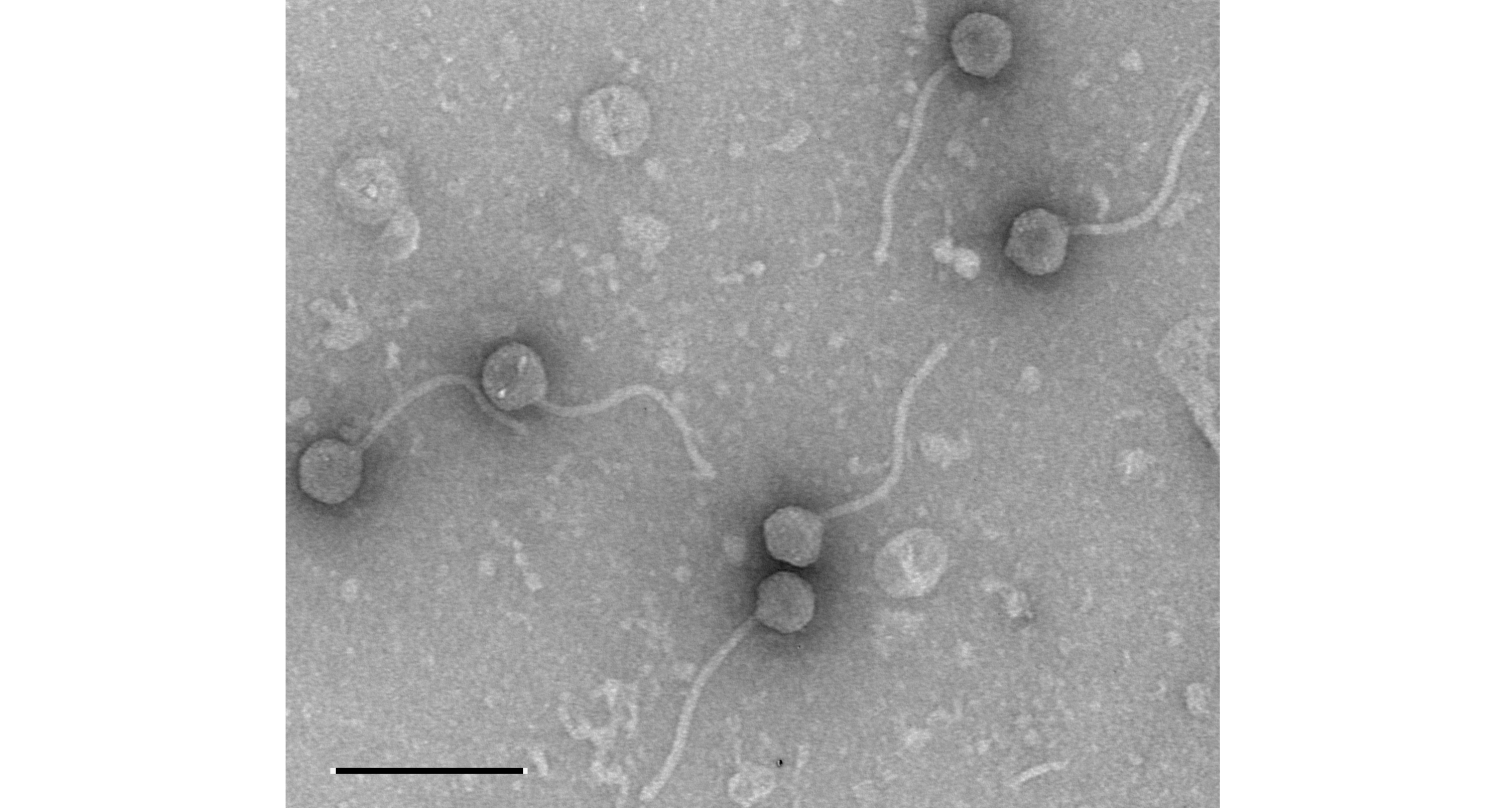
Negative-stained (1% uranyl acetate) transmission electron microscopy of virions of bacteriophage Wheezy exhibit a siphoviral morphology. Measurements of six particles showed tail lengths ranging from 231 to 249 nm (average 238 nm) and 54 to 62 nm diameter capsids (average 59 nm). Scale bar = 200 nm. Image acquired at the Nebraska Center for Biotechnology Morrison Microscopy Core Facility (Univ. Nebraska-Lincoln) using Hitachi Model HT-7800.

## Description

Members of the Gram-positive *Gordonia* genus, in the Actinobacteria phylum, are found largely in soil and aquatic environments (Sowani et al., 2018) , and include a number of pathogenic isolates (Ramanan et al., 2013; Gulvik et al., 2021) . Capable of diverse metabolic transformations, *Gordonia* species are reservoirs of genes that can be exploited for bioremediation and industrial biotechnology (Arenskötter et al., 2004; Drzyzga, 2012; Sowani et al., 2019) . Bacteriophages infecting *Gordonia* are potential molecular tools for studying the genes coding for metabolic enzymes of *Gordonia* (Dyson et al., 2015) . We report the genome sequence of Wheezy, an actinobacteriophage infecting the soil bacterium *Gordonia terrae* 3612.

Wheezy was obtained from a soil sample collected from Black Elk Park (Millard, NE; GPS 41.198411 N, 96.168891 W) using *G. terrae* 3612 as the host following standard protocols for enriched isolation (Zorawik et al., 2024) . Briefly, soil was mixed with peptone-yeast extract-calcium (PYCa) liquid media and the collected extract was filter-sterilized with a 0.22-μm filter before inoculation with *G. terrae* 3612. After incubation at 30˚C, with shaking for 24 hours, the extract was centrifuged to remove bacteria and tested for phage using *G. terrae* 3612 in soft (PYCa) agar plaque assays, with plates being incubated for 48 h at 30˚C. Wheezy made clear plaques of 1 to 1.5 mm diameter. After three rounds of plaque purification, negative-stained Wheezy phage from a lysate were observed by transmission electron microscopy to have a siphoviral morphology ( Figure 1 ).

Phage DNA was isolated from a lysate using the Wizard DNA Cleanup Kit (Promega Corp., Madison, WI), prepared for sequencing with the New England Biolabs Ultra II Library Kit and sequenced at the Pittsburgh Bacteriophage Institute using Illumina MiSeq (v3 reagents). A total of 563,642 (150-base single-end) reads provided 1257-fold coverage. Genome assembly was accomplished and checked for sequence quality and completeness with Newbler v2.9 and Consed v29 (Russell, 2018) .

The Wheezy genome was 67,021 bp with a 3’ single-stranded overhang (CGCCGCGTAC) and 65.9% GC, and assigned to cluster CR (subcluster CR2) based on gene content similarity (GCS) of 35% or higher with phages in the Actinobacteriophage database (phagesdb.org), using the database GCS tool (Pope et al., 2017; Russell and Hatfull 2017) . Wheezy was most similar to GrootJr, sharing 98.38% of its phams (phage protein families) followed by NovumRegina and NatB6, sharing 97.83% and 97.3%, respectively, of their phams with Wheezy (Russell, 2018) .

The genome of Wheezy was automatically annotated with DNA Master v5.23.6 (Lawrence, 2007) that used Glimmer v3.92b (Delcher et al., 2007) and GeneMark v2.5p (Besemer and Borodovsky 2005) to identify open reading frames (ORFs). Neither tRNAs nor transfer-messenger RNAs (tmRNA) were detected using the programs tRNAscan-SE 2.0 and ARAGORN v1.2.41 (Lowe and Eddy 1997; Laslett and Canback 2004) . Web-based bioinformatic analysis software programs including NCBI BLASTp (Altschul et al., 1997) using NCBI non-redundant protein sequences and Actinobacteriophage Protein databases, Starterator (phages.wustl.edu/starterator/) and HHPred 3.18 (Soding et al., 2005) , using PDB mmCIF70, SCOPe70, Pfam-A and NCBI Conserved Domain databases, were used to confirm or revise start sites and assign functions for 40 of a total of 92 ORFs. These include six membrane proteins identified using Deep TMHMM v 1.024 (Hallgren et al., 2022) and SOSUI v1.11 (Hirokawa et al., 1998) . All tools were run with default parameters. Gene maps of Wheezy and other CR2 subcluster phage (genomes average 92 total genes) were compared using Phamerator (Actino_Draft v513) (Cresawn et al., 2011) .

The left arm of the Wheezy genome encodes genes that are transcribed in the forward direction (gp1–51). Genes for the terminase large subunit, and phage structural and assembly proteins, including the tail assembly chaperones that are predicted to be produced through a translation frameshift (Xu et al., 2004) , are clustered together (gp24–44). As seen in CR Cluster phage, the two domains of lysin A are encoded by adjacent but separate genes (gp47 and gp48). Genes transcribed in the reverse direction are located roughly in the middle portion of the genome (gp52–77) and include ones predicted to encode proteins that interact with DNA such as WhiB family transcription factors (gp54 and gp73), Dna-E like DNA polymerase III (gp60) and DNA helicase (gp72). The remaining downstream genes are transcribed in the forward direction and encode proteins with unknown functions (gp78–92). Genes for neither integrase nor repressor functions could be identified, suggesting that the phage is unlikely to establish lysogeny.

**Nucleotide sequence accession numbers**

Phage Wheezy is available at GenBank with Accession No. OR475262 and Sequence Read Archive (SRA) No. SRX20630260 .
